# Multimodal Field-Driven Actuation in Bioinspired Robots: An Emerging Taxonomy and Roadmap Towards Hybrid Intelligence

**DOI:** 10.3390/biomimetics10100713

**Published:** 2025-10-21

**Authors:** Jianping Wang, Xin Wang, Shuai Zhou, Gengbiao Chen

**Affiliations:** 1 Advanced Vocational Technical College, Shanghai University of Engineering Science, Shanghai 200437, China; wangjianping@sues.edu.cn; 2 College of Mechanical and Vehicle Engineering, Changsha University of Science and Technology, Changsha 410114, China; 23203030740@csust.edu.cn (X.W.); zhoushuai@stu.csust.edu.cn (S.Z.)

**Keywords:** biohybrid actuation, stiffness modulation, multifield coupling, embodied intelligence, rigid–flexible coupling

## Abstract

Rigid–flexible coupled robots hold significant potential for operating in unstructured environments, but a systematic analysis of their actuation strategies across diverse physical fields is notably lacking in the literature. This review addresses this gap by introducing a novel taxonomy based on field-controlled evolutionary pathways—mechanical → electromagnetic → chemical → biohybrid—and critically examining over 100 seminal studies through a six-dimensional framework encompassing design, dynamics, and performance. We demonstrate that hybrid field integration (e.g., pneumatic-chemical synergy) improves grasping robustness by 40% in cluttered environments compared to single-field systems. Notably, biohybrid actuators, which integrate living cells, exhibit over 90% motion similarity to biological models, while phase-transition materials allow for adaptive stiffness tuning (0.1–5 N·mm^−1^) in medical applications. Radar chart analysis further reveals fundamental trade-offs between energy efficiency, response speed, and scalability across the various fields. These insights provide a clear roadmap for the development of next-generation robots with embodied intelligence, emphasizing multi-field synergies and bio-inspired adaptability.

## 1. Introduction

**Figure 1:**
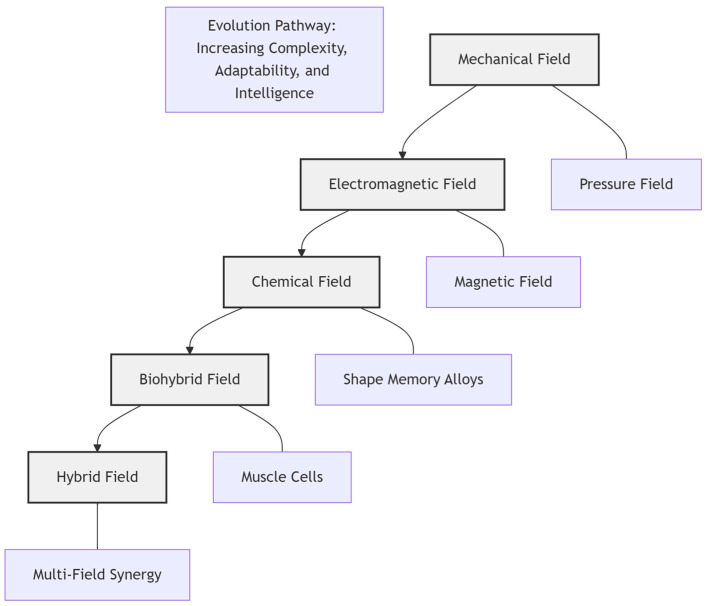
Taxonomy visualization diagram. Author’s original work.

The integration of rigid and compliant components has emerged as a critical paradigm in robotics, enabling systems that combine the precision of traditional robots with the adaptability and safety inherent to soft systems. These rigid–flexible coupled robots have found applications across a range of industries, driven by pressing societal needs. In industrial manufacturing [1,2,3], they are key to achieving flexible automation and remanufacturing in the context of evolving product cycles and labor shortages. In medical surgery [4,5,6], they address the demand for less invasive procedures and personalized rehabilitation in aging societies. Their use in wearable robotic systems [7,8,9] responds to the need for enhanced human augmentation and assistive technologies. The advancement of these robots is therefore not merely a technological pursuit but a response to global socioeconomic trends [10,11,12,13,14,15]. Existing reviews often focus on singular materials or actuation types, neglecting the interdisciplinary convergence of fields that is driving the development of this domain. To address this gap, we propose a novel, field-driven taxonomy. This framework categorizes rigid–flexible coupling robots into five primary categories based on their energy field: mechanical, electromagnetic, chemical, biological, and mixed-field effects. This taxonomy and its proposed evolutionary pathway—from mechanical → electromagnetic → chemical → biohybrid → hybrid fields—are visualized in Figure 1. The diagram not only categorizes the actuation strategies but also illustrates a logical progression toward systems with greater embodiment and intelligence, which is further elaborated in the subsequent sections. For each category, we analyze the system characteristics, implementation strategies, and relevant application areas, while also projecting future directions for the development of rigid–flexible coupling mechanisms.

To systematically evaluate the performance differences among various driving modes, this paper establishes a six-dimensional evaluation framework. It quantitatively assesses the systems across six core dimensions: load capacity, accuracy, response speed, energy consumption, environmental adaptability, and control complexity.

## 2. Classification Development and Framework Validation Methods

This review uses a systematic literature review method to develop and validate the proposed Domain Driven classification and evaluation framework. This process ensures that the classification is based on existing research and that the evaluation criteria can be consistently applied to the available data in the published literature.

### 2.1. Systematic Literaturer Retrieval and Selection

In the main scientific databases (IEEE Xplore, web of science, Scopus), key words related to “rigid flexible robot”, “soft robot drive”, “bionic drive” and specific domain names (such as “pneumatic”, “magnetic drive”, “shape memory alloy”) are used for comprehensive search. The search range is from 2010 to 2024. The research contents include: (1) a complete robot system with rigid flexible coupling characteristics is proposed; (2) Provide quantitative performance data for at least two of the six evaluation dimensions. More than 100 studies that meet these criteria form the basis of this analysis.

### 2.2. Data Extraction and Standardization Protocol

In order to ensure the consistency evaluation of different studies, a standardized data extraction scheme was developed.

Performance data extraction: for each reviewed system, quantitative data (e.g., maximum force, response time, positioning error) is extracted directly from tables, charts or text descriptions in the original publication.

Unit standardization: convert all extracted data into standard international units (SI units) for cross study and comparison (for example, mm represents accuracy, n represents load capacity, and seconds represents response speed).

Threshold based scoring: then use the predefined quantitative threshold in Table 1 to map the standardized data to the 1–5 scoring scale. For parameters that are not explicitly reported (e.g., control complexity), perform a systematic analysis based on the control architecture described in the literature, taking into account the number and types of sensors, the presence and nature of feedback loops, the complexity of control algorithms, and the level of autonomy, among other factors, to draw conclusions. For example, a system described as “open-loop control using a single on/off command” is rated 1; a system using single-sensor-based closed-loop control is rated 2; a system with fixed and linear control logic is rated 3; a system employing model-based, adaptive, or intelligent control strategies, including impedance control, neural networks for compensating nonlinearity, or controllers capable of adjusting parameters online based on system dynamics or environmental changes, is rated 4; and systems involving biological signal fusion or advanced autonomous decision-making are rated 5.

### 2.3. Inter Rater Reliability Assessment

In order to verify the objectivity of the scoring process, the two authors independently evaluated a subset of 30 representative studies using the same extraction scheme and evaluation criteria.

## 3. Rigid–Flexible Coupling Robots with Mechanical Field Actuation

Rigid–flexible coupling technologies driven by mechanical fields primarily operate through pressure fields, flow fields, and vibration fields. Their development follows a progressive trajectory of bionic optimization → functional integration → environmental adaptation. Early research drew inspiration from biological adhesion mechanisms—such as octopus suction cups—and gradually evolved from single-function gripping to adaptive operation in complex environments via pneumatic networks and composite structural designs. For example, negative-pressure adsorption technology has addressed the challenge of balancing contact stability with energy efficiency, progressing from basic suction cup designs to advanced composite suction cups. Similarly, vibration-field actuation combines rigid transmission with flexible friction through soft-foot collaborative designs, significantly enhancing mobility across diverse terrains. This progression reflects the systematic evolution of mechanical field-driven systems, transitioning from bionic prototypes to integrated engineering solutions.

### 3.1. Pressure Field in Rigid–Flexible Coupling

**Figure 2:**
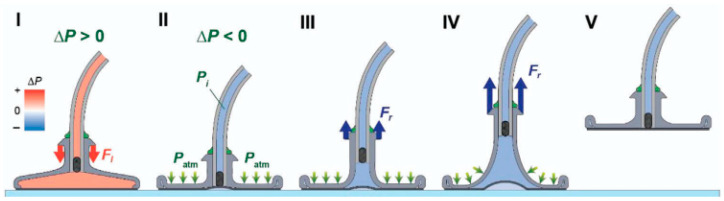
The principle of action of the suction cup. (**I**) The gripper contacts the substrate under the action of preload; (**II**) A negative pressure difference is applied internally, pulling up the middle part of the film and forming an initial suction cavity; (**III**) The gripper starts to retract, and the soft cavity deforms, exposing more areas of the film to negative pressure and leading to the passive expansion of the suction cavity. (**IV**) Under the maximum pull-off force, the cavity deformation intensifies, presenting a three-leg structure while the seal remains intact; (**V**) The film is peeled off from the contact edge, the seal fails, and air leakage causes the suction to disappear. Adapted from [19], licensed under CC BY 4.0.

Inspired by the adhesive properties of octopus suckers, several researchers have explored the use of negative pressure within gas–liquid cavities to generate suction and thrust for object retention [16,17,18]. A representative example is the cup clamp introduced by Sukho Song (Figure 2) [19]. In its initial configuration, the gas–liquid cavity remains hermetically sealed, preserving a relatively rigid state that stabilizes both shape and hardness. This rigidity enables precise alignment with the target object. When manipulation is required, pneumatic pressure is adjusted within the cavity, shifting the structure from a rigid to a compliant state and allowing the robot to envelop the object effectively. Such systems demonstrate high efficiency in grasping objects in cluttered or unstructured environments.

However, conventional suction cups face inherent limitations, particularly under non-ideal contact angles or insufficient surface contact forces. To address these issues, Sensen Liu and colleagues developed a composite suction cup comprising an inner soft cup and an outer rigid cup [20]. The inner soft cup provides superior adaptability, accommodating angular mismatches and maintaining a secure seal against irregular surfaces. Meanwhile, the outer rigid cup enhances adhesion strength, thereby improving stability and adsorption efficiency. Experimental evaluations show that this composite suction cup sustains approximately 90% adsorption efficiency even under external disturbances such as crosswinds, markedly enhancing its robustness in complex real-world scenarios.

**Figure 3:**
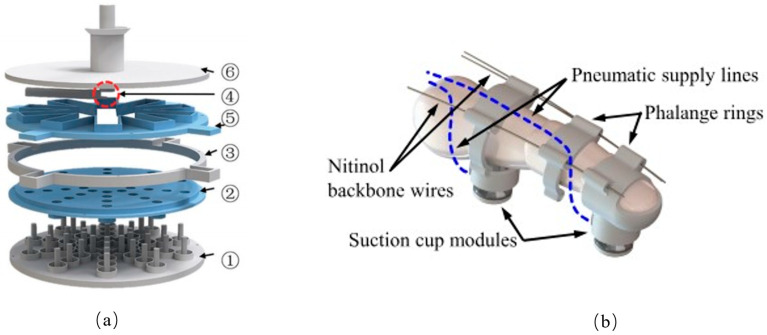
(**a**) A disc-shaped rubber soft body with multiple suction cups for adaptable grasping of small and large objects. ①, ②: Suction orifice molds, responsible for forming the funnel-shaped suction orifice at the bottom of the suction cup and the internal vertical channel; ③, ④: Channel wall molds, assembled with parts 1 and 2, jointly forming the side walls of the internal annular cavity connecting each suction orifice; ⑤, ⑥: Core molds, serving as support structures, used to shape the complex interconnected horizontal main channels inside the suction cup during the casting process. They can be removed and reused during demolding. Adapted from [21], licensed under CC BY 4.0. (**b**) Suction cups mounted on a hand exoskeleton gimbal to expand the grasping range for users with hand impairments. Adapted from [22], licensed under CC BY 4.0.

Many researchers have focused on integrating suction cups into robotic gripper systems to enhance object grasping capabilities. For instance, Mingxin Wu proposed a gripper design that combines a 3D-printed connecting rod with a soft suction cup (Figure 3a) [21], while Seokhwan Jeong developed a hand exoskeleton utilizing a self-sealing suction cup module (Figure 3b) [22]. In another design [23], suction cups are arranged on a disc-shaped rubber soft body. For small objects, the suction cup and rubber disc work in tandem: the suction cup facilitates adhesion, while the rubber disc contracts to secure the object. For larger objects, the suction cup alone performs the grasping function. Additionally, in a design proposed by [24], suction cups are mounted on the gimbal of a hand exoskeleton, allowing for a wider reach. This hand exoskeleton assists individuals with hand impairments by expanding their grasping capabilities. However, the system’s reliance on a larger vacuum tank and air pump can limit arm mobility.

Despite the advantages, key challenges remain in the use of suction cup manipulators, particularly in controlling suction force to avoid object damage and ensuring stable contact force during grasping. These issues continue to be active areas of research.

**Figure 4:**
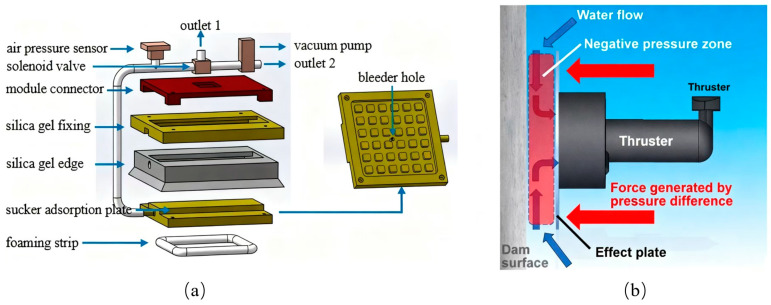
(**a**) A suction cup integrated into the robot’s plantar surface with closed-loop pressure control for stable climbing. Adapted from [23], licensed under CC BY 4.0. (**b**) A suction cup base on a dam inspection robot for reliable adhesion in dynamic underwater environments. Adapted from [25], licensed under CC BY 4.0.

Beyond object manipulation, suction cups play a critical role in stabilizing a robot’s relative position. For example, Chen Xue integrated a suction cup into the robot’s plantar surface (Figure 4a), creating a closed-loop control system that continuously monitors real-time data from a barometric pressure sensor [25,26]. This integration significantly enhanced the robot’s stability and adaptability during climbing operations. Similarly, Norimitsu Sakagami placed a suction cup at the base of a dam inspection robot (Figure 4b), enabling reliable performance in dynamic underwater environments while conducting inspection tasks both at the dam’s base and surrounding aquatic areas [27,28].

### 3.2. Flow Field Actuation in Rigid–Flexible Coupling

**Figure 5:**
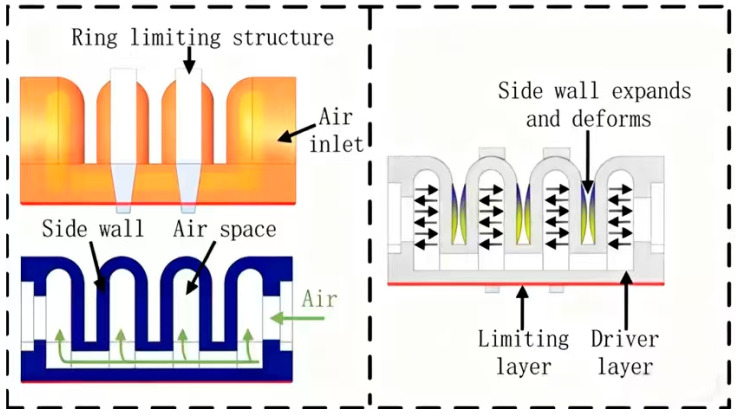
Mechanism of action of fast pneumatic network. Adapted from [40], licensed under CC BY 4.0.

Numerous manipulators based on flow field principles leverage rapid pneumatic networking approaches [29,30,31,32,33,34,35,36], as illustrated in Figure 5, where multiple air cells are interconnected. When external air pressure is applied to these cells, the arm of the air chamber expands and deforms. However, the bottom plate features a non-retractable restraint layer, which causes the entire actuator to bend. In the absence of external pressure, the actuator remains flexible, allowing for adaptable operation. Conversely, as air pressure within the chamber increases, the actuator becomes more rigid, thereby enhancing its support and precision in positioning.

**Figure 6:**
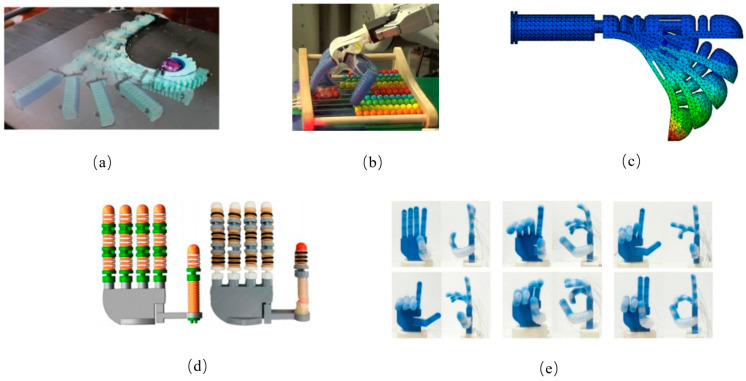
Rigid–flexible coupling method based on flow field. (**a**) Octopus-inspired multi-cavity soft manipulator for non-destructive grasping. Adapted from [37], licensed under CC BY 4.0. (**b**) Soft hand with reduced control complexity via imitation learning. Adapted from [38], licensed under CC BY 4.0. (**c**) Biomimetic flexible finger with jointed pipes for increased load capacity. Different colors represent the stress and strain of the fingers. Adapted from [39], licensed under CC BY 4.0. (**d**) Thumb with omnidirectional movement paired with modular soft fingers. Adapted from [40], licensed under CC BY 4.0. (**e**) Multi-cavity manipulator for gesture expression and multi-target grasping. Adapted from [41], licensed under CC BY 4.0.

Several studies have utilized rapid pneumatic network approaches for object grasping. For instance, the flexible plane clamping manipulator (Figure 6a), proposed by Robert K. Katzschmann’s team in 2015 [37], achieved non-destructive grasping of flat objects by a soft robot for the first time, achieving a grasping success rate of over 90%. This was accomplished using a multi-cavity design and a visual positioning system that mimics the structure of an octopus hand, serving as a key example for pneumatic network-driven systems. However, limitations became evident in subsequent studies. First, the grasping system relies on static target positioning, making it ineffective for handling moving objects or surfaces with disturbances. Second, the purely flexible structure of the air cavity limits its load capacity (with a maximum load of <500 g) and lacks a dynamic stiffness adjustment mechanism. Lastly, the system is restricted to flat-object grasping, making it unsuitable for unstructured 3D environments.

These limitations were addressed through suction cup-gimbal integration. For example, the soft hand (Figure 6b) developed by Abhishek Gupta’s team [38] reduced control complexity (training time was reduced by 50%) through an imitation learning algorithm. However, the system’s single-degree-of-freedom pneumatic finger achieves a low success rate (32%) in fine operations, such as pinching objects with diameters under 1 mm. The primary issue is the absence of haptic feedback and stiffness adaptation mechanisms.

In response, Jiten Sun’s team proposed a biomimetic flexible finger (Figure 6c) design that mimics human finger joints, replacing traditional fingers with jointed pipes and pneumatic modules to replicate human-like bending and grasping capabilities. This design increases load-bearing capacity compared to conventional soft robotic fingers [39]. However, their work focused solely on the fingertip, neglecting the integration of the finger into a complete palm mechanism, which limits its ability to assess comprehensive grasping performance.

Building upon this, Chang Chen’s team developed a thumb with omni-directional movement (Figure 6d), paired with four modular soft fingers made from a rigid base material, overcoming the limitation of single-directional grasping inherent in traditional planar flexible manipulators [40]. Unfortunately, this design is still limited to static grasping.

Tianze Hao’s manipulator (Figure 6e) [41] enabled gesture expression and multi-target grasping through a 14-cavity design, but its lack of lateral movement freedom makes it unsuitable for industrial tasks such as rotation and tightening. Additionally, its aerodynamic delay (response time > 0.5 s) limits its application in dynamic environments. In contrast, fast pneumatic networks, as reported in Ref. [30], reduce this delay to 0.2 s. While Hao’s design facilitates the expression of sign language gestures and the grasping of objects with various sizes, shapes, weights, and textures, it lacks lateral opening and closing movements, restricting its ability to perform more complex tasks.

Lastly, Andrade-Silva introduced a compact, foldable five-pointed star-shaped soft clip that is portable when not in use. However, this design requires specific size and hardness parameters for the target object [42].

Table 2 summarizes the evolutionary trajectory of rigid–flexible coupling based on flow field dynamics, emphasizing significant advancements in integrating rigid joints with pneumatic network knuckles. This integration improves the structural integrity of finger support, enabling more precise manipulation of objects in three-dimensional space. However, a fundamental limitation of this type of manipulator remains: it is typically restricted to basic actions such as grasping or simulating human movements, which limits its ability to perform more complex tasks. To address this limitation, Xinjie Zhang introduced a novel manipulator that combines pneumatic network gripping with suction cup fastening, resulting in a stronger grip suitable for more intricate and advanced operational contexts [43]. To deploy a rigid flexible coupling robot in a large-scale industrial environment, its full life cycle cost and environmental footprint must be considered. At present, many soft robots rely on polymer materials such as silica gel, which have poor recyclability and may bring new challenges of electronic waste. From the perspective of energy, the energy consumption of the continuously running pneumatic pump is huge, which is contrary to the goal of low-carbon industry, so it is urgent to develop energy-saving driving scheme. In addition, the economy of technology adoption is the key: although the flexible gripper can adapt to a variety of products, its life may not be as long as the traditional steel gripper, and enterprises need to weigh its flexibility and replacement and maintenance costs. Future technological development should incorporate the principle of circular design, giving priority to repairability, upgradeability and recyclability.

### 3.3. Vibration Field Actuation in Rigid–Flexible Coupling

Vibration-driven robots interact with substrates by generating inertial and elastic forces through oscillatory motion. These forces cause the robot’s compliant “soft feet” to engage in frictional contact with the surface, resulting in slippage that produces a propulsive effect. This mechanism exemplifies the synergy between rigid propulsion systems and compliant foot designs—an essential feature of vibration-field-driven rigid–flexible coupling.

The vibrating robot developed by Satoshi Iyobe’s team [44] demonstrated multiple motion modes in a single-module system by adjusting the motor’s vibration frequency. On flat surfaces, the robot exhibited excellent deformation capabilities. However, the reliance on a single-beam element structure limited its task complexity, while stability was significantly reduced in environments containing multiple obstacles. Furthermore, the absence of contextual awareness modules prevented real-time terrain adaptation.

Building on locomotion strategies, Yuyang Zhao’s earthworm-inspired robot [45] achieved superior mobility in narrow pipes through phase-difference control between adjacent modules. Despite this efficiency, its locomotion was restricted to the horizontal direction, with little capacity for vertical climbing. Additionally, rigid connections between modules reduced flexibility, making it prone to jamming in bent pipelines.

**Figure 7:**
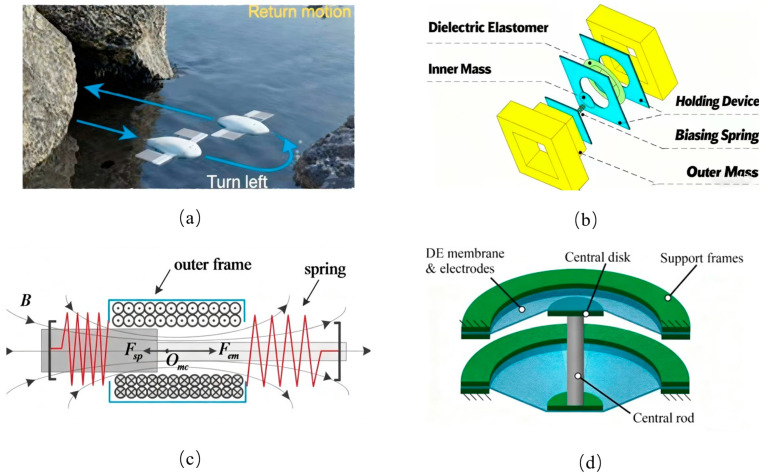
Rigid–flexible coupling structure based on the vibration field. (**a**) Amphibious robot using flexible fins and soft feet. Adapted from [46], licensed under CC BY 4.0. (**b**) Robot activated by periodic stiffness modulation. Adapted from [47], licensed under CC BY 4.0. (**c**) Compact mobile robot with two parallel oscillators. Adapted from [49], licensed under CC BY 4.0. (**d**) Vibrating robot powered by an elastic dielectric actuator. Adapted from [50], licensed under CC BY 4.0.

To address multi-environment locomotion, Dehong Wang introduced an amphibious vibration-driven robot (Figure 7a) [46]. This design transferred motor vibrations directly to the environment, enabling locomotion on water via flexible fins and on land via soft feet. The absence of a traditional transmission mechanism made the robot compact, lightweight, and efficient. However, these advantages came at the expense of versatility in more complex operational tasks.

Similarly, Xiaojian Wang proposed a robot activated by periodic stiffness modulation (Figure 7b) [47]. By inputting a sine waveform and controlling vibration frequency, the robot was able to move both forward and backward in isotropic friction environments. Although the system demonstrated simple control and low environmental demands, it lacked the versatility required for complex maneuvers.

Van-Du Nguyen developed an innovative vibration-driven capsule robot module [48], achieving bidirectional motion by oscillating an internal mass using half-sinusoidal electromagnetic forces. While effective under isotropic friction conditions, discrepancies were observed between theoretical predictions and experimental outcomes.

Xiong Zhan introduced a compact vibration-driven mobile robot (Figure 7c) [49]. Using two parallel oscillators for propulsion, the design generated anisotropic friction with the ground via blade-like soft feet. By adjusting vibration frequency and amplitude, the robot achieved two-dimensional movement. Nevertheless, its locomotion was constrained by limited precision and efficiency.

Finally, Chuang Wu proposed a novel vibrating robot powered by an elastic dielectric actuator (Figure 7d) [50]. Unlike traditional actuators based on servo motors or spiral coils, this actuator responded directly to electric field stimuli, enabling high driving strain, flexibility, and cost-effectiveness. Despite these advantages, the system was limited to normal environments and unsuitable for adhesive or highly complex surfaces.

### 3.4. Comprehensive Comparison of Mechanical Field Actuation

The performance analysis of rigid–flexible coupling robots driven by mechanical fields (summarized in Table 3) evaluates each representative system based on structural characteristics and task performance.

The composite suction cup (negative-pressure adsorption) employs an inner–outer cup composite structure and achieves rigid–flexible state switching by regulating the pressure within a gas–liquid cavity. This design maintains adsorption efficiency above 90% in disordered environments [20], earning a load capacity score of 4.0. Closed-loop pressure sensing stabilizes the contact force, limiting positioning errors to within 10 mm [23], resulting in an accuracy score of 3.5. However, pneumatic response delays of 100–500 ms [22] limit its responsiveness (3.0 points). Continuous operation requires a vacuum pump to maintain negative pressure, yielding high energy consumption (2.5 points) [22]. Thanks to its dual-structure design, the system demonstrates excellent resilience in complex conditions such as crosswind interference [20], with an environmental adaptability score of 4.5. Control relies on pneumatic valve switching and sensor feedback, with relatively low difficulty in coordinating multiple degrees of freedom, resulting in control complexity of 2.0 [23].

The flexible planar clamping manipulator (pneumatic network) achieves non-destructive grasping through a multi-chamber design inspired by the octopus hand, with an accuracy of 4.0 points [34]. However, its purely flexible structure limits load-bearing capacity (maximum < 500 g, rated 3.0 points) [34]. Response speed is comparable to that of the composite suction cup (3.0 points) [34], while energy consumption remains similar (2.5 points) [22]. Its application scope is restricted to two-dimensional planes, giving it only moderate environmental adaptability (3.0 points) [34]. The integration of a visual positioning system increases algorithmic complexity, raising control complexity to 3.5 [34].

The vibration-driven amphibious robot transmits inertial vibration forces via flexible fins and soft feet. It demonstrates strong adaptability in amphibious environments, with an obstacle-crossing height of ≥20 mm and an inclination angle of ≥30° [41], earning environmental adaptability of 4.5 points. Its dynamic load capacity is 2.3 times higher than that of traditional flexible fingers [39], meriting a 3.5-point load score. However, trajectory deviations of about 5% [49] reduce its accuracy to 2.5 points. Pneumatic delays (>0.5 s) [41] constrain its responsiveness (3.0 points). On the positive side, the absence of a complex transmission mechanism enhances efficiency, with energy consumption rated at 3.0 [46]. Control depends mainly on motor frequency adjustments, with relatively few parameters, resulting in control complexity of 2.5 [41].

Overall, mechanical field actuation demonstrates strong load capacity and excellent environmental adaptability, making it effective in scenarios such as industrial grasping and multi-terrain locomotion. However, its performance remains constrained by high energy consumption and response speed bottlenecks inherent to pneumatic and vibration-based systems [2,22,34,41,46,49].

### 3.5. Practical Deployment and Scalability Challenges

The mechanical field driver, especially the pneumatic system, has excellent performance in the laboratory environment, but there are inherent bottlenecks in its industrial promotion. Low energy efficiency is a core problem: in order to maintain negative pressure or drive the pneumatic network, it is usually necessary to carry bulky air pumps or vacuum tanks, which greatly limits the autonomy and working time of mobile platforms [22,34]. Secondly, the system response speed is limited by gas compressibility and pipeline delay, which is difficult to meet the needs of high-speed automatic production. In addition, the reliability will be significantly reduced in harsh industrial environments (such as dusty and large temperature difference), and the durability of the software structure still needs to be verified for a long time. To realize the leap from laboratory to industry, future research needs to focus on developing compact and efficient power sources, improving material durability and designing robust control algorithms that can adapt to the uncertainty of the real environment.

## 4. Rigid–Flexible Coupling Robots with Electromagnetic Field Actuation

The development of electromagnetic field-driven technology follows an observable progression pattern—from electric fields to magnetic fields and then to light fields—that can be explained by principles from systems theory. This progression reflects a fundamental trend in engineering systems: the drive toward increased ideality (achieving more functionality with fewer resources) and enhanced controllability. Electric field-based systems (e.g., traditional motors) provide high power but limited precision. Magnetic field systems enable non-contact manipulation at smaller scales, while light field actuation offers the ultimate in remote, wireless control. This evolution mirrors the TRIZ principle of “increasing dynamism and controllability” in technological systems. This framework reflects the underlying logic of technological advancement: through progressive optimization of field characteristics, increasingly efficient and precise control capabilities can be realized

Early research focused on the electromechanical coupling of electroactive polymers (EAPs), in which electric fields directly induced material deformation. This laid the physical foundation for electromagnetic field-driven robotics. With growing demands for miniaturization, magnetic field-driven systems have demonstrated unique advantages in medical micro–nano operations, providing non-contact manipulation and precise positioning without bulky mechanical structures. More recently, the emergence of light-field actuation has addressed limitations in energy supply. Light-responsive materials such as liquid crystal gels enable remote, long-duration operation, advancing robotic systems toward higher autonomy.

### 4.1. Rigid–Flexible Coupling Under Electric Field Actuation

Electric-field-driven actuation methods can be broadly categorized into two approaches:

Motor-based actuation—the most widely used method [51]. Here, rigid–flexible coupling arises from the interaction between a rigid actuator and its compliant housing.

Electroactive polymer (EAP)-based actuation—in which electrification induces structural transformations. In the unenergized state, polymer films remain flexible without morphological change. Upon energization, electrostatic forces between conductive layers cause deformation and bending, enabling the film to transition into a stiffer, load-bearing configuration. This approach is valued for its high strain capacity, low weight, and remarkable flexibility, making it well-suited for robotic applications.

**Figure 8:**
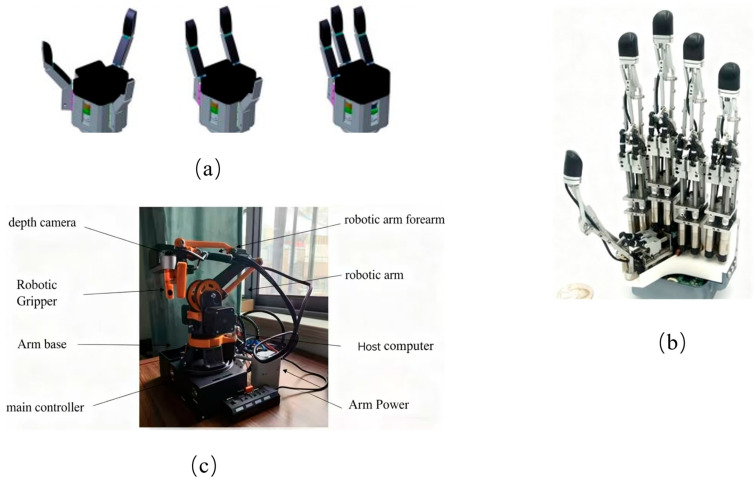
Rigid–flexible coupling mode based on the motor. (**a**) Multi-finger manipulator with strong driving force. Adapted from [52], licensed under CC BY 4.0. (**b**) A 19-joint robotic system with postural coordination. Adapted from [54], licensed under CC BY 4.0. (**c**) Highly flexible four-axis robotic arm. Adapted from [55], licensed under CC BY 4.0.

Current research in this area is largely concentrated on motor-driven manipulators. Representative contributions include the following:

Shufeng Tang’s multi-finger manipulator (Figure 8a) [52]—a rigid–flexible coupling design offering stronger driving forces compared to pneumatic systems. It adapts gripping strategies to different scenarios, enhancing both safety and functionality.

Patricia Capsi Morales’s 19-joint robotic system [53]—integrates postural coordination into motion control, significantly improving flexibility, safety, and adaptability in object handling.

Uikyum Kim’s chain-architecture robotic hand (Figure 8b) [54]—integrates actuation and sensing components, providing 15 degrees of freedom. Its hollow structure reduces weight while incorporating tactile sensing, enabling seamless integration with robotic arms for versatile gripping tasks.

Yijie Wang’s four-axis robotic arm (Figure 8c) [55]—characterized by high flexibility and intelligence, capable of performing tasks beyond conventional robotic arms.

Caidong Wang’s ankle-assist rehabilitation robot [56]—despite only having two degrees of freedom, it supports three distinct movement modes and effectively reduces secondary injuries during therapy.

Xiaoyu Wang’s dynamic model of spatial curved slip mechanisms [57]—demonstrates that nonlinear characteristics of flexible components reduce the system’s effective elastic modulus, thereby lowering joint reaction forces in rigid–flexible coupling systems.

**Figure 9:**
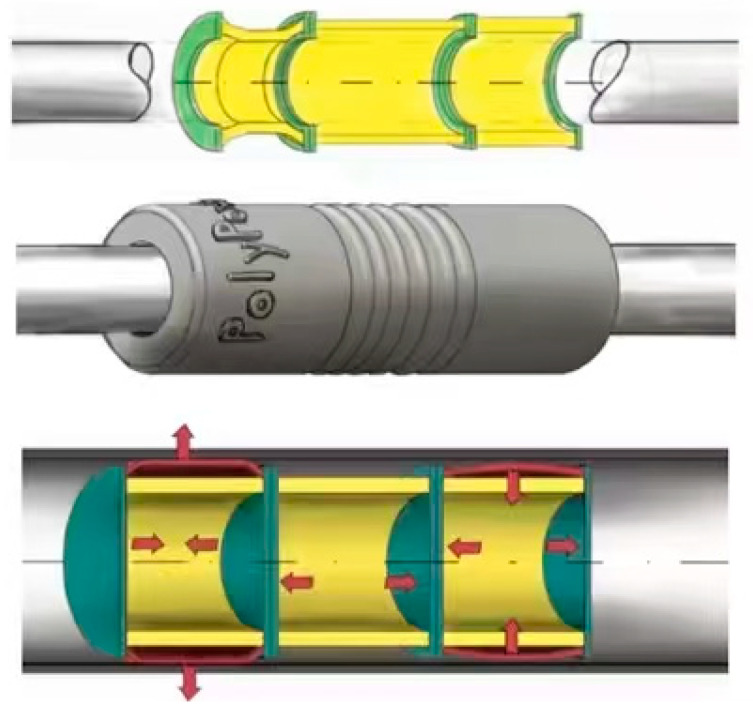
Rigid–flexible coupling based on electroactive polymers. From top to bottom, the cross-sectional view of the actuator crawling outside the cylindrical rod, the overall appearance of the actuator, and the cross-sectional view of crawling inside the cylindrical pipe are shown respectively. The red arrows clearly indicate the main movement direction of each DEAP actuator when it is energized and activated. Adapted from [58], licensed under CC BY 4.0.

Compared with conventional motor-driven actuators, electroactive polymer (EAP) actuators have recently emerged as a major research focus owing to their superior flexibility. Singh proposed a novel three-layer elastomer structure resembling a sandwich configuration (Figure 9) [58]. In this design, the top and bottom layers function as electrodes, typically fabricated from materials such as carbon grease, graphite, carbon nanotubes, or carbon black. The middle layer consists of a dielectric elastomer, commonly made of silicone, acrylic, or polyurethane. When a high voltage is applied, electrostatic stress between the electrodes compresses the dielectric layer, causing the actuator to deform. Once the voltage is removed, the intrinsic elasticity of the dielectric restores the actuator to its original shape.

Further advancing theoretical understanding, Noy Cohen analyzed the deterministic behavior and phenomenological models of three electromechanical configurations of EAPs under uniform deformation, examining four distinct boundary conditions [59]. This comparative study provided deeper insights into the predictive accuracy and limitations of existing modeling approaches for EAP-based actuators.

### 4.2. Rigid–Flexible Coupling Under Magnetic Field Actuation

Magnetic actuators are widely employed in biomedical research due to their capacity for free spatial arrangement and their ability to enter living organisms without obstruction. In one experimental study [60], each magnetic fiber contained thousands of programmable magnetically responsive structures that could be precisely controlled to bend or stretch. Within the rigid–flexible coupling framework, the fibers remain compliant in the absence of a magnetic field, but upon field application, they undergo controlled bending or stretching, thereby transitioning into a more rigid state. Leveraging this property, spiral-gap magnetic robots can transport small payloads during deformation, enabling microrobot-based drug delivery. Similarly, guidewires can be designed with tunable magnetic properties to enable precise bending, enhancing their utility for tasks such as targeted lesion removal.

**Figure 10:**
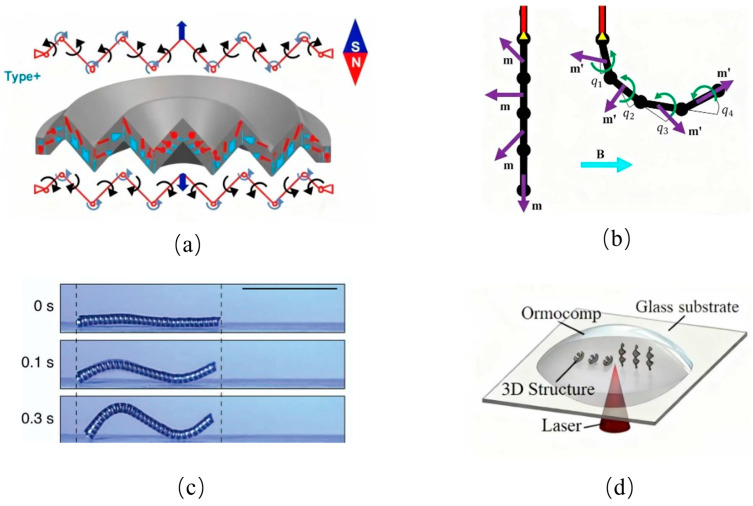
Rigid–flexible coupling based on magnetic field. (**a**) Magnetic actuation film for rapid deformation. Adapted from [61], licensed under CC BY 4.0. (**b**) Magnetically actuated soft robot with winding and stretching motions. The left side shows the initial reference state, displaying the discrete links and their preset magnetization vectors (m). Under the action of an external magnetic field (B), the joints rotate, the magnetization vectors change to (m*), and the deformation is driven by magnetic torques (indicated by green arrows). Adapted from [63], licensed under CC BY 4.0. (**c**) Spiral-gap magnetic fiber for payload transport. Adapted from [64], licensed under CC BY 4.0. (**d**) Spiral capsule-shaped microrobot for reduced intestinal friction. Adapted from [65], licensed under CC BY 4.0.

The magnetic actuation film (Figure 10a) developed by Dezhao Lin’s team [61] achieves rapid deformation through radial magnetization, providing a lightweight and responsive actuation solution for soft robots. However, its driving force is limited, and the material is prone to fatigue under high-frequency magnetic excitation. Furthermore, the accuracy of magnetic control is easily affected by environmental interference [62]. Despite these limitations, its fast response and low weight make it well-suited for soft robotic applications.

Peter Lloyd introduced a novel magnetically actuated soft robot (Figure 10b) [63], which employs a uniform magnetic field in combination with a skeletal structure to achieve controlled winding and stretching. Unlike Youngbin Lee’s spiral-gap fiber transport method (Figure 10c) [64], which moves objects within fiber cavities, Lloyd’s design enables direct winding-based manipulation, making it especially promising for long-distance drug delivery inside the human body.

Expanding on capsule microrobotics, Yunlong Jia developed a spiral capsule-shaped microrobot (Figure 10d) [65], composed of a spiral body, an O-ring neodymium magnet, a shaft, and an outer shell. By modulating the magnetic field generated by three Helmholtz coils, the microrobot’s spiral jet propulsion can be precisely tuned in both speed and frequency. This reduces intestinal friction and improves performance in confined biological environments.

Finally, Yicheng Ye designed a sub-millimeter magnetron soft microrobot [66], featuring a 1 mm diameter PDMS rod-shaped beam coupled with two N52 neodymium magnets. To improve guidewire maneuverability in medical applications, a spring mechanism was incorporated, allowing the PDMS bundle to integrate with sub-millimeter catheter guidewires. This design offers enhanced precision in minimally invasive navigation.

Although the magnetic capsule robot has shown great potential for accurate navigation in the laboratory, its clinical transformation is still full of non-technical obstacles. Regulatory approval is a major bottleneck: such systems, which integrate machinery, magnetism and software, belong to a brand-new category of medical devices, and need to establish brand-new safety and effectiveness evaluation standards. This process may take several years and is expensive. Data privacy and security are also crucial, because real-time video and data transmission in patients must comply with the most stringent medical data protection regulations (such as gdpr and HIPAA). In addition, whether the medical insurance payment system will pay for this new technology will directly determine its accessibility and commercialization success. Therefore, future research must have a dialogue with regulators, clinicians and medical insurance payers as soon as possible to jointly design a feasible transformation path.

### 4.3. Rigid–Flexible Coupling Under Light Field Actuation

The effectiveness of electric and magnetic field actuation is inherently constrained by signal attenuation, which limits their operational duration. In contrast, light energy—whether from sunlight or artificial sources—offers robots the potential for extended operational lifetimes [67,68].

**Figure 11:**
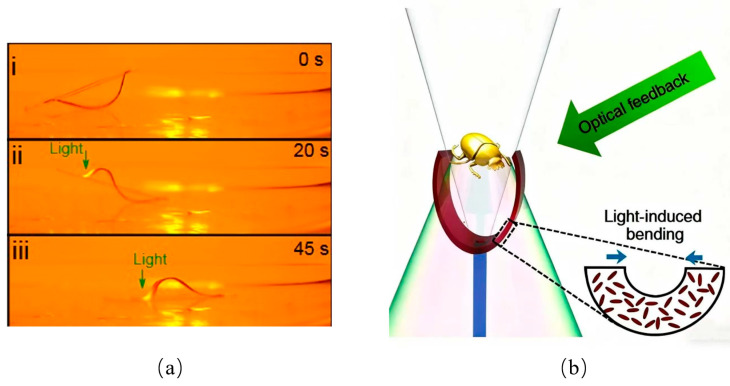
Rigid–flexible coupling based on light field. (**a**) Rigid–flexible coupling of optical drive during swimming phase. (i) Initial state: The LCG-50 structure is in a relaxed state; (ii) Illumination phase: 532 nm laser scanning induces local bending, forming a propagating wave of undulation that pushes the unexposed part forward; (iii) Center-of-mass transfer: Elastic potential energy is released, and the entire structure displaces forward, completing one motion cycle. Adapted from [69], licensed under CC BY 4.0. (**b**) Light-driven flytraps. Adapted from [70], licensed under CC BY 4.0.

M. Pilz da Cunha investigated the unique properties of photochromic liquid crystal gel (LCG) materials and their ability to alter morphology through light absorption (Figure 11a) [69]. These materials employ photopolymerization to selectively separate locked liquid crystal molecules, thereby initiating macroscopic deformation. This work demonstrated the feasibility of solar-powered robotic systems, while also underscoring the challenge of achieving more complex and diverse motion patterns. Within the rigid–flexible coupling framework, LCGs remain compliant in the absence of illumination, but upon light exposure they deform and transition into a more rigid configuration, completing the flexible-to-rigid transformation.

Shahsavan advanced this research by developing a monolithic LCG with molecular anisotropy (Figure 11b) [70]. Under controlled illumination, the material can induce localized deformations, mimicking the adaptive movements of mollusks and aquatic invertebrates. This property provides a powerful basis for biomimetic robotic applications. Notably, the smooth transition between rigidity and flexibility demonstrated by this material establishes a robust foundation for the development of purely light-driven robots.

Wani introduced a novel light-driven flytrap design [71], employing light-responsive liquid crystal elastomers as the actuation medium. These materials efficiently absorb reflected and scattered ambient light, triggering deformation that powers the actuation process. The flytrap is further enhanced by an integrated feedback mechanism, enabling autonomous adjustment of light-induced forces. This design illustrates a high degree of intelligence and adaptability, marking an important step toward self-sustaining, light-driven robotic systems.

### 4.4. Comprehensive Comparison of Electromagnetic Field Actuation

The performance analysis of electromagnetic field–driven rigid–flexible coupling robots (summarized in Table 4) evaluates representative cases according to field control characteristics and task adaptability.

The magnetically controlled capsule robot [65] achieves precise spiral motion by adjusting the magnetic field generated through three Helmholtz coils. Its positioning error is less than 10 mm, and its response delay is under 10 ms, corresponding to an accuracy score of 5.0 and a response speed score of 4.5. However, the payload capacity is limited to micro-drug loads (<5 g), resulting in a load capacity rating of 2.0. Since it relies on external magnetic field sources for power, energy efficiency is moderate (3.5 points) [65]. Additionally, magnetic field penetration in biological tissue is constrained to <15 cm, reducing its environmental adaptability (3.0 points). The system also requires a three-dimensional magnetic positioning algorithm for spatial pose estimation, yielding a control complexity score of 3.5.

Electroactive polymer (EAP) fingers exploit the electromechanical properties of dielectric elastomers, achieving millisecond-level deformation responses [58], with a response speed score of 4.5. The sandwich-layered design further improves fingertip positioning accuracy to 0.5 mm, earning an accuracy score of 4.0. However, due to the material’s elastic limit, the maximum gripping force is only 0.3 N, resulting in a load capacity score of 2.5. With no need for complex transmission mechanisms, EAP fingers demonstrate moderate energy efficiency (3.5 points) [58]. Nonetheless, they are highly sensitive to humidity (performance degrades above 60% RH), which reduces their environmental adaptability to 3.0. Control is based on high-voltage regulation with relatively few parameters, leading to a control complexity rating of 3.0.

The light-driven liquid crystal gel (LCG) robot achieves morphological changes by unlocking light-induced molecular rearrangements. Its energy conversion efficiency reaches 15% [69], corresponding to an energy score of 4.0. With light intensity gradient positioning, the robot achieves a 3D trajectory error below 2%, earning an accuracy score of 4.0 [70]. However, its load-bearing capacity is very limited (<1 g), rated at 1.5 points. The response time is restricted by light penetration (1–3 s), giving a response speed score of 3.5. In addition, the system requires clean and controlled lighting environments (e.g., cleanrooms), resulting in low environmental adaptability (2.5 points). Control complexity is high due to the need for synchronous regulation of wavelength, intensity, and irradiation angle, resulting in a score of 4.0 [69,70].

Overall, electromagnetic field actuation demonstrates clear advantages in accuracy and response speed, especially for precision micro–nano robotic applications. However, its load capacity and environmental adaptability remain constrained by field penetration limits and the intrinsic properties of active materials [58,65,69,70].

### 4.5. Challenges and Industrial Application Bottlenecks

Although the electromagnetic field drive has excellent performance in accuracy and response speed, it faces a series of fundamental challenges when it moves from precision laboratory to large-scale industrial application or deployment in unrestricted environment.

First, the penetration depth and attenuation of the field are inherent physical limitations. Whether it is magnetic field, electric field or optical field, its intensity and controllability will decline sharply with the increase in distance. For example, the effective working distance of magnetic navigation system in biological tissues is usually limited to 10–15 cm [65], which greatly limits its operating range in large organisms or complex industrial equipment. For light driven robots, their applications are strictly limited to transparent or translucent media, because light cannot penetrate most opaque materials [69,70].

Secondly, energy supply and thermal management are the key reliability bottlenecks. The equipment that produces high-intensity and high-precision magnetic field or electric field is usually bulky and energy consuming. At the same time, the high-frequency electromagnetic field will induce eddy current loss in the conductor, resulting in local overheating of the driver, which may damage the equipment or surrounding tissues in long-term operation or medical applications [58,66]. Although the optical drive can realize wireless power supply, the high-energy laser itself is not a portable device, and there are security risks.

Thirdly, environmental disturbance and system complexity restrict its robustness. The electromagnetic field is easily disturbed by metal objects in the environment, resulting in the decline of control accuracy. In the industrial field, the complex electromagnetic environment makes the operation of micro nano robots based on weak magnetic or weak electric signals almost impossible. In addition, complex coordination of multiple external field generators (such as three sets of Helmholtz coils) is often required to achieve multi-dimensional accurate control, which leads to high cost, high complexity and low accessibility of the system [65].

Finally, the durability of materials is also a major concern. The risk of dielectric breakdown of electroactive polymers under high voltage, the demagnetization of magnetic materials and the aging of photosensitive materials all affect the service life of electromagnetic field drive systems in industrial scenarios requiring long-term stable operation.

To sum up, the future breakthrough of electromagnetic field drive not only needs to continue to optimize the performance of the material itself, but also depends on making leaps and bounds in portable field generator, anti-interference control algorithm and efficient thermal management strategy to overcome its inherent physical and application limitations.

## 5. Chemically Field-Based Rigid–Flexible Coupling Robots

### 5.1. Rigid–Flexible Coupling Under Chemical Field Interaction

At the core of chemical field actuation lies the dynamic response of smart materials—such as shape memory alloys (SMAs)—to external chemical or thermal stimuli. The technological evolution of this field follows the trajectory of “material innovation → functional expansion → biocompatibility.” Early research on SMAs centered on thermally induced deformation, providing the foundation for rigid–flexible switching. Subsequent work combined microstructural design with biocompatibility, extending their application to minimally invasive surgery and biomimetic motion simulation. More recent efforts have focused on optimizing self-healing SMAs with improved cell compatibility, pushing chemical field actuation from engineering devices toward implantable medical systems.

Shape memory alloys (SMAs) represent a cutting-edge class of smart materials distinguished by their high sensitivity to thermal stimuli and unique shape memory properties. Unlike conventional alloys, SMAs can return to their original form upon exposure to temperature fluctuations, a feature that has attracted substantial attention in materials science [72,73,74,75]. For robotic applications, SMAs offer a distinct advantage: in the absence of heat, the structure remains flexible and adaptive, but upon heating, it rapidly transitions into a rigid state, enabling versatile rigid–flexible coupling. These properties have made SMAs an essential material for clinical robotics.

**Figure 12:**
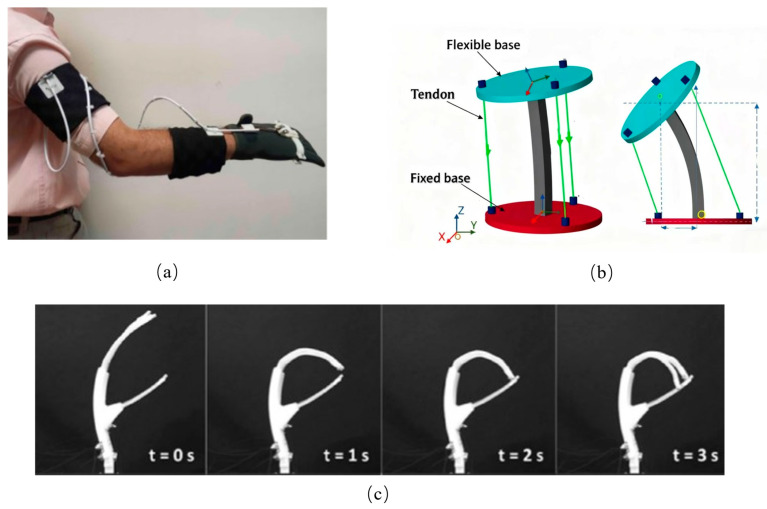
Rigid–flexible coupling based on chemical field. (**a**) Lightweight SMA soft actuator for free bending. Adapted from [76], licensed under CC BY 4.0. (**b**) SMA-driven soft neck with dual-degree-of-freedom Adapted from [79], licensed under CC BY 4.0. (**c**) Hinged artificial finger combining SMA with smart soft composites. Adapted from [80], licensed under CC BY 4.0.

For instance, Seyedreza Kashef Tabrizian designed an SMA-based bending brake (Figure 12a) [76], capable of autonomously performing closure and self-healing without human intervention. This innovation significantly enhances the intelligence and automation of medical devices, though further optimization is required to address complex surgical demands.

Beyond SMAs, two-dimensional transition metal carbides/nitrides (MXenes) [77] have emerged as promising candidates for smart robotics. Produced by selectively etching strongly bonded layered precursors, MXenes exhibit diverse electronic, optical, chemical, and mechanical properties, opening new avenues for soft robotics. In his comprehensive review, Yizhou Wang emphasized their potential for next-generation robotic materials [78].

Dorin-Sabin Copaci advanced SMA applications by proposing a lightweight soft actuator design (Figure 12b) [79]. This actuator maintains high flexibility while enabling free bending, making it suitable for rehabilitation devices and artificial bone applications. Copaci further developed an SMA-driven soft neck (Figure 12c) [80], achieving human-level tilt and steering accuracy through a dual-degree-of-freedom design. Its quiet operation offers an advantage for rehabilitation, but SMA hysteresis causes trajectory deviations and limits driving frequency, restricting real-time interaction. Moreover, its maximum torque (<0.3 Nm) is insufficient to support exoskeleton-assisted loads.

Hehua Zhang developed a biocompatible SMA spiral microrobot capable of modulating torque and thrust through morphological adaptation [81]. This device demonstrates excellent motion performance and long-term operational potential, making it highly suitable for minimally invasive surgery. Similarly, Hyung-Il Kim’s team introduced a hinged artificial finger combining SMAs with smart soft composites [82], replicating human-like bending and grasping. Although currently limited to light object manipulation, it shows significant promise for improving robotic dexterity.

Expanding biological simulation, Xiaonan Huang created an SMA-based soft robot capable of mimicking diverse motions such as human walking and caterpillar crawling [83]. This highlights the adaptability of SMA robots in complex environments, though more advanced movements (e.g., jumping or aquatic locomotion) remain future challenges. Similarly, Hyun-Taek Lee’s team designed a miniature SMA-embedded tentacle robot [84], capable of achieving strong gripping forces on small objects, with potential applications in surgical target fixation. However, miniaturization introduces inefficient heat dissipation, and SMA biocompatibility remains unproven. Additionally, the requirement for high-temperature actuation poses risks of tissue damage in biomedical applications.

### 5.2. Comprehensive Comparison Driven by Chemical Field

The performance analysis of chemical field–driven rigid–flexible coupling robots (as shown in Table 5) evaluates each case based on material response characteristics and adaptability to application scenarios.

The SMA bending brake achieves structural rigidity switching through temperature-triggered martensitic transformation. Its spiral structure generates a torque of 2 N·mm [76], resulting in a load capacity rating of 3.0. The system achieves bending angle accuracy with a less than 5° error [76], yielding an accuracy score of 3.0. However, the phase transition requires 2–5 s to complete, limiting its response speed rating to 2.5. Powered by a lithium battery (150 Wh/kg energy density), energy consumption during intermittent operation is rated 4.0 [76]. It operates stably in the range of −20 °C to 80 °C, achieving a environmental adaptability rating of 3.0. The system’s control logic is based on temperature threshold triggering, resulting in a control complexity score of 2.5.

The MXene soft actuator uses two-dimensional material catalytic reactions to generate deformation-driving forces, but its interlayer sliding mechanism limits the maximum output force to 1.2 N [78], leading to a load capacity score of 2.5. The reaction process is sensitive to solution concentration fluctuations, with positioning errors up to 8% [78], resulting in an accuracy score of 2.5. The catalytic reaction delay ranges from 3 to 8 s, reducing the response speed rating to 2.0. No external energy is required as the actuator relies on chemical reaction energy storage, earning an energy consumption rating of 4.0 [78]. However, the actuator oxidizes easily in humid environments, with a lifespan of less than 100 cycles, giving it an environmental adaptability score of 2.5. Coordinating material ratio and reaction rate introduces control complexity, resulting in a complexity rating of 3.0 [78].

The biological hybrid SMA fingers combine skeletal muscle cell contraction and SMA actuation to achieve a gripping force of 0.8 N [82], leading to a load capacity score of 2.0. Electromyographic feedback is used to adjust gripping force, with a force control error of less than 12% [82], resulting in an accuracy rating of 3.5. However, the cell response delay (1–2 s), combined with SMA thermal hysteresis, limits the overall response speed to 2.5. The cellular metabolic energy supply contributes 30% of the total energy, giving it a comprehensive energy consumption rating of 4.5 [85]. These hybrid fingers can survive in a biological culture medium for ≥48 h, achieving an environmental adaptability rating of 3.5. The need to synchronously regulate cell activity, temperature fields, and mechanical feedback results in a control complexity rating of 4.0 [82,85].

Chemical field actuation offers significant advantages due to the adaptive properties of material phase transitions and its potential for low energy consumption. However, its slow response speed and biocompatibility challenges limit its applicability in dynamic tasks [76,78,82,85].

## 6. Future Development Trend of Rigid–Flexible Coupling Methods

### 6.1. Biofield-Based Rigid–Flexible Coupling Robots

Most current rigid–flexible coupling structures rely on single-field actuation modes—such as mechanical, electromagnetic, or chemical fields. According to the Technological Development Trend, these systems are expected to progress toward biofield-based robots and eventually toward multi-field hybrid actuation. Early progress in this direction has already been observed.

Initial studies of biofield-driven actuation focused on mimicking natural locomotion by employing living cell drives such as muscle cells [86,87,88]. However, these systems were limited by insufficient driving forces. Recent approaches have combined biological components (e.g., light-driven bacteria [89]) with synthetic materials, enabling multi-degree-of-freedom (DOF) motion and greater task complexity. Future research is anticipated to move toward neural interfaces and biological feedback systems, advancing toward genuine “bio-mechanical symbiosis” through deep human–machine integration.

One of the persistent challenges in prosthetics is that many amputees still perceive prostheses as external tools rather than extensions of their bodies, largely due to limitations in miniaturization and realism [90]. Consequently, biocompatibility has become a critical factor in robotic development. Since many biological cells inherently generate force [91], the use of biological actuators—combined with efficient microstructural designs—could replicate natural motor behaviors. Biohybrid systems, derived from living tissue, inherently provide flexibility, safety, and compatibility with human–computer interaction, making them highly attractive for medical robotics.

For instance, skeletal muscle cells can resume contraction upon electrical stimulation, providing a novel means of driving soft materials for deformation and locomotion. Kazuma Morita developed a biohybrid actuator composed of C2C12 cells and fibrin-based hydrogels in a tensile monolithic structure [85]. Unlike conventional muscle actuators—typically limited to planar single-DOF motion—this design employs a 3D multi-tissue configuration, achieving multi-DOF movements. Similarly, Nicola Pellicciotta proposed a microrobot powered by optically driven bacteria, enabling intricate micro-scale tasks [92].

Advancing the integration of biosignals, Andrés Úbeda designed a manipulator combining computer vision and surface electromyography (sEMG) [93]. The system could evaluate pre-grasping postures via computer vision and refine them using sEMG signals, improving grasping performance by 13%. Likewise, Minkyu Shin’s team embedded muscle bundles into a PDMS substrate, achieving multi-DOF locomotion in biohybrid actuators [94]. Despite these advances, practical deployment remains restricted by low muscle-cell driving forces, challenging culture conditions, and the high costs and long timelines of cell preparation.

Efforts in prosthetics and neuroprosthetics also highlight these challenges. Retheep Raj developed a biomechanical model of the human forearm to analyze motion dynamics [95], while Susannah M. Engdahl assessed user experience with body-powered versus myoelectric prostheses [96], finding that current EMG-based devices offer limited improvements in user perception. This underscores the need to move beyond surface EMG toward neural interface–based prosthetics for truly intuitive control.

For example, Melanie G. Urbanchek created a peripheral nerve regeneration interface for neuroprosthetic control, serving as both a stable biological interface and a signal amplifier [97]. Meanwhile, Ryuki Kinjo’s team developed a skeletal muscle cell–driven biped robot, achieving fine gait control with a ±0.1 mm step accuracy by optimizing skeletal muscle contractility and robot geometry [98]. Although this represents a major advance in miniaturization and motion coordination, the robot still depends on external electrical stimulation and requires continuous nutrient supply, limiting its autonomy.

In another breakthrough, Noriyasu Ando’s group designed a biohybrid odor-tracking robot, integrating Bombyx mori silkworms with a micromechanical platform [99]. Unlike traditional actuators, this system leveraged the natural chemotaxis of silkworms, transmitting antennal nerve signals via implantable electrodes to guide robotic navigation. While this represents a novel paradigm of “living organism–mechanical synergy,” survival time was limited by physiological stress from electrode implantation, and odor sensitivity declined when exposed to non-natural stimuli.

In biomedical applications, safety and immune rejection remain critical challenges. Addressing these concerns could unlock broad opportunities for biofield-based rigid–flexible coupling robots, particularly in prosthetics, neuroprosthetics, and therapeutic devices.

### 6.2. Rigid–Flexible Coupling Robots with Mixed-Field Actuation

Most rigid–flexible coupling robots rely on a single field for actuation; however, this approach has inherent limitations when applied in isolation. For example, pneumatic systems typically require large air pumps to enable gripping, which significantly restricts their operational range. To address such constraints, researchers are investigating whether gas volume changes in pneumatic networks can be induced by alternative fields. Accordingly, mixed-field-driven rigid–flexible coupling robots are expected to become a major research direction, and several studies have already demonstrated their potential.

Wu et al. [100] introduced a lightweight soft crawling robot based on negative pressure adsorption. This robot achieves dynamic adsorption and detachment through a light-controlled gas–liquid phase transformation. In this design, the suction cup integrates both pressure and light fields, underscoring the promise of mixed-field coupling. Its simple fabrication and compact structure make it particularly suitable for miniaturized soft robots and adaptable to diverse soft robotic applications.

Similarly, Pan et al. [101] developed a soft-body cavity detonation driver that regulates hydrogen and oxygen content within an air cavity. The controlled hydrogen–oxygen mixture undergoes combustion, producing an explosive force that drives a gas-powered jumping robot. The robot’s jumping capability arises from the combined effects of the flow field and the chemical field, exemplifying how multi-field interactions can enable unique robotic motions.

### 6.3. Comprehensive Comparison Between Biological Field and Hybrid-Field Actuation

Table 6 presents a performance comparison of rigid–flexible coupling robots driven by biological fields and mixed fields. The evaluation criteria include the characteristics of biological components and the efficiency of multi-field coordination.

The muscle-cell PDMS actuator achieves three-dimensional structural deformation through the contraction of C2C12 skeletal muscle cells, with a maximum contraction stress of 0.1 mN per cell [85]. However, due to cell density limitations, its load-bearing capacity lasts only 1.5 min. Electrical stimulation can regulate the phase of cell contraction, achieving over 90% similarity with biological motion trajectories, corresponding to an accuracy rating of 4.0. The coupling delay between excitation and contraction is approximately 2 s, resulting in a response speed rating of 1.0. Powered by glucose metabolism (energy conversion efficiency ≈ 18%), it receives a maximum score of 5.0 for biological-level energy consumption. Nonetheless, maintaining functionality requires a constant temperature of 37 °C and a sterile environment, limiting environmental adaptability to 2.0. Moreover, coordinating cell differentiation, electrode arrangement, and mechanical feedback imposes high control complexity, rated at 5.0 [85].

The light-controlled negative-pressure adsorption robot integrates light-field-driven gas–liquid phase transitions with pressure-field adsorption. It exhibits a rapid light-triggered phase transition of 0.2 s [100], earning a response speed rating of 3.5. Its composite suction cup enhances suction force up to 20 N, with a load capacity score of 3.5. Closed-loop control of adsorption and release via light intensity yields positioning errors below 3 mm, corresponding to an accuracy score of 3.5. Compared with single-pressure actuation, the light–air synergy reduces energy consumption by 40%, earning a rating of 3.0. The system remains functional under both illuminated and non-illuminated conditions, with an environmental adaptability score of 4.0. However, activation of the light–gas coupling control algorithm introduces moderate control complexity, rated at 4.0 [100].

The odor-tracking hybrid robot employs silkworm moth antenna neural signals for navigation, achieving biological olfactory localization accuracy of ±5 mm in odor-source detection [99], corresponding to an accuracy rating of 3.0. Energy supply relies on moth metabolism (≈0.1 mW), achieving the highest efficiency rating of 5.0. However, the survival time of silkworm moths is less than 12 h, reducing environmental adaptability to 3.0. Furthermore, decoding neural signals and mapping them to mechanical movements requires advanced bio-signal analysis and control strategies, resulting in a control complexity rating of 5.0 [99].

Overall, biological-field actuators demonstrate disruptive potential in energy efficiency and biomimetic adaptability. Nevertheless, the fragility of biological components and the complexity of hybrid-field algorithms continue to hinder their practical deployment [91,99,100].

### 6.4. Challenges and Ethical Considerations

Although the bio hybrid actuator shows great potential in energy efficiency and biocompatibility, its practical application still faces severe challenges. First of all, biosafety is the primary concern: the escape of living cells, the environmental release of genetically modified organisms and the risk of pathogen transmission must be strictly controlled. Secondly, ethical issues need to be discussed openly: the integration of neural tissues or sensory organs into machines has raised profound questions about machine consciousness, life dignity and moral status. In addition, the actual deployment obstacles are huge: cell culture requires complex life support systems (such as sterile environment and constant temperature nutrition supply), which seriously limits the running time and range of motion of the robot. Finally, the low driving force of cells and the difference between batches lead to unstable performance, which is far from industrial reliability. Therefore, future research must put the development of ethical framework and large-scale life support system in the same important position as technological breakthrough.

## 7. Comparison of Various Drives

Mechanical-field drives demonstrate strong performance in load capacity (4.0 ± 0.5) and environmental adaptability (4.5 ± 0.3). For example, composite suction cups achieve efficient adsorption through structural optimization [20], while vibration-based robots maintain stability across diverse terrains [41]. However, their energy efficiency remains limited (2.5 ± 0.2) due to dependence on pumps.

Electromagnetic-field drives are particularly suitable for precision applications, such as magnetic capsule robots that achieve positioning errors below 10 mm [65]. They offer high accuracy (4.5 ± 0.8) and fast response speed (4.5 ± 0.5). Nevertheless, their environmental adaptability is relatively low (3.0 ± 0.7), as performance is constrained by magnetic field penetration and susceptibility to electromagnetic interference or light pollution.

Chemical-field drives are recognized for favorable energy efficiency (4.0 ± 0.3), exemplified by the intermittent heating mode of shape-memory alloys (SMA). Yet, their response speed (2.5 ± 0.6) is limited by the intrinsic delay of material phase transitions.

Biological-field drives achieve biological-level energy efficiency (5.0 ± 0.0), but their load capacity (1.5 ± 0.3) and response speed (1.0 ± 0.2) are restricted by cell activity and viability [85].

**Figure 13:**
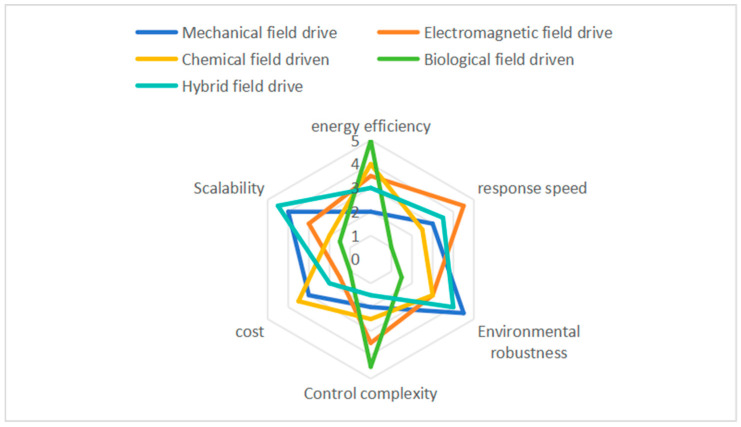
Radar images driven by different fields. Author’s original work.

Hybrid-field drives improve environmental adaptability (4.0 ± 0.3) through strategies such as light–air coupling [100]. However, their control complexity (4.0 ± 0.3) is significantly higher due to the need for cross-field collaborative algorithms. The completed radar analysis chart is shown in Figure 13.

The performance dispersion (confidence intervals) across different drive types reflects their technological maturity. Mechanical and electromagnetic drives exhibit relatively low fluctuations, indicating stable and well-developed engineering applications. By contrast, biological and chemical drives show high variability, suggesting that further advancements in materials and control algorithms are required to enhance reliability and consistency.

## 8. Conclusions

This review establishes a field-driven taxonomy that traces the evolution of rigid–flexible coupling technologies, highlighting a clear progression from single-field actuation toward multifield and biohybrid integration. Despite notable advances, two overarching challenges continue to limit widespread adoption: (i) inherent trade-offs among performance metrics (e.g., speed versus load capacity), and (ii) the integration complexity of hybrid systems.

Future breakthroughs are expected to emerge through the development of biofield- and mixed-field-based actuation strategies. Biofield-inspired models may draw from natural biological systems, such as the cooperative dynamics of the musculoskeletal system, to enable smoother and more efficient transitions between rigid and flexible states. In parallel, mixed-field models are anticipated to harness the complementary strengths of different actuation fields, thereby supporting the creation of robots with enhanced functionality, adaptability, and autonomy.

Ultimately, advancements in multi-field coupling and intelligent control will lay the foundation for the next generation of robots, capable of operating with greater efficiency, resilience, and autonomy across diverse application scenarios. These insights provide a clear roadmap for the development of next-generation robots with embodied intelligence. However, the ultimate success of these technologies will be measured not only by their technical prowess but by their ability to address grand societal challenges—from providing compassionate care for an aging population to enabling sustainable manufacturing—in a safe, ethical, and equitable manner. Future research must, therefore, embrace a transdisciplinary approach that integrates technical innovation with rigorous consideration of regulatory, environmental, and social implications.

## Figures and Tables

**Table 1:** Six-dimensional evaluation standard table.

Dimension	Grading Criteria (1 to 5 Points)	Unit/Description
Load capacity	1: <0.1 N; 2: 0.1–1 N; 3: 1–10 N; 4: 10–50 N; 5: >50 N	Based on maximum output force or graspable weight
Precision	1: >20 mm; 2: 10–20 mm; 3: 1–10 mm; 4: 0.1–1 mm; 5: <0.1 mm	Positioning error or repeated positioning accuracy
Response speed	1: >5 s; 2: 1–5 s; 3: 0.1–1 s; 4: 10–100 ms; 5: <10 ms	Time from command to action completion
Energy consumption	1: Continuous high power consumption; 2: Intermittent high power consumption; 3: Medium efficiency; 4: Low power consumption; 5: Self-powered/biological energy level	Energy efficiency ratio or power demand
Environmental adaptability	1: Laboratory only; 2: Limited environment; 3: General indoor; 4: Multi environment; 5: Extreme/complex environment	Including temperature and humidity, medium, interference, etc.
Control complexity	1: Open-loop simple control; 2: Closed-loop single parameter; 3: Multi-sensor feedback; 4: Multi-field coupling control; 5: Biological signal fusion	Control algorithm, number of parameters, system integration

**Table 2:** Comparison of rigid–flexible coupling methods based on flow field.

Author	Year	Problem Solved	Merit	Shortcoming
Robert K. Katzschmann [37]	2015	Object Gripping	No damage to the object; Highly dexterous	Can only be used on 2D planes
Abhishek Gupta [38]	2017	Simulation of Human Hands	Low cost; More precise movements	Precise movements not fully achievable
Jiten Sun [39]	2022	Finger Simulation	High fingertip load capacity	No linkage with the palm
Chang Chen [40]	2022	Complete Design of Entire Robot	Multi-directional grip	Dynamic crawling not possible
Tianze Hao [41]	2023	Complex Finger Movements	High flexibility	Complex movements not fully achievable

**Table 3:** Comparison of mechanical field performance.

Drive Type	Load Capacity	Accuracy	Response Speed	Energy Consumption	Environmental Adaptability	Control Complexity
pressure field	4.0	3.5	3.0	2.5	4.5	2.0
flow field	3.0	4.0	3.0	2.5	3.0	3.5
vibration field	3.5	2.5	3.0	3.0	4.5	2.5

**Table 4:** Comparison of Electromagnetic Field Performance.

Drive Type	Load Capacity	Accuracy	Response Speed	Energy Consumption	Environmental Adaptability	Control Complexity
magnetic field	2.0	5.0	4.5	3.5	3.0	3.5
electric field	2.5	4.0	4.5	3.5	3.0	3.0
light field	1.5	4.0	3.5	4.0	2.5	4.0

**Table 5:** Comparison of chemical field performance.

Drive Type	Load Capacity	Accuracy	Response Speed	Energy Consumption	Environmental Adaptability	Control Complexity
SMA bending brake	3.0	3.0	2.5	4.0	3.0	2.5
MXene soft actuator	2.5	2.5	2.0	4.0	2.5	3.0
Biological hybrid SMA finger	2.0	3.5	2.5	4.5	3.5	4.0

**Table 6:** Comprehensive comparison between biological field and mixed growth.

Drive Type	Load Capacity	Accuracy	Response Speed	Energy Consumption	Environmental Adaptability	Control Complexity
Muscle cell PDMS actuator	1.5	4.0	1.0	5.0	2.0	5.0
Light controlled negative pressure adsorption robot	3.5	3.5	3.5	3.0	4.0	4.0
Odor tracking biological hybrid robot	1.0	3.0	2.0	5.0	3.0	5.0

## Data Availability

Data are available from the corresponding author upon reasonable request.
